# Making room for life and death at the same time – a qualitative study of health and social care professionals’ understanding and use of the concept of paediatric palliative care

**DOI:** 10.1186/s12904-022-00933-4

**Published:** 2022-04-11

**Authors:** Anette Winger, Elena Albertini Früh, Heidi Holmen, Lisbeth Gravdal Kvarme, Anja Lee, Vibeke Bruun Lorentsen, Nina Misvær, Kirsti Riiser, Simen A. Steindal

**Affiliations:** 1 Department of Nursing and Health Promotion, Faculty of Health Sciences, Oslo Metropolitan University, P.O. Box 4, St. Olavs plass, NO-0130 Oslo, Norway; 2 Oslo University Hospital, Oslo, Norway; 3 VID Specialized University, Oslo, Norway; 4 Lovisenberg Diaconal University College, Oslo, Norway

**Keywords:** Concept, Paediatric, Pediatric, Palliative care, Terminal care, PPC, Life limiting, Alleviation, Health care professionals

## Abstract

**Background:**

The concept of pediatric palliative care (PPC) is applied differently within the healthcare system and among healthcare professionals (HCPs). To our knowledge, no studies have investigated how multidisciplinary HCPs understand the concept of PPC and the aim of this study was to explore the concept of PPC from the view of HCP in a paediatric setting.

**Methods:**

We employed an explorative and descriptive design and conducted four focus groups with a total of 21 HCPs working in hospitals with children in palliative care. The data were analysed using qualitative content analysis.

**Results:**

The data analysis of the concept of pediatric palliative care resulted in two themes. The first theme “A frightening concept that evokes negative emotions,” contains categories to explore the meaning, named “An unfamiliar and not meaningful concept, “A concept still associated with death and dying” and “Healthcare professionals’ responsibility for introducing and using the concept and, to obtain a common meaning.” The second theme was named “A broad and complementary concept,” containing the categories “Total care for the child and the family,” “Making room for life and death at the same time” and “The meaning of alleviation and palliative care.”

**Conclusions:**

The included HCPs reflected differently around PPC but most of them highlighted quality of life, total care for the child and the child’s family and interdisciplinary collaboration as core elements. Attention to and knowledge among HCPs might change the perception about PPC from a frightening concept to one that is accepted by all parties, implemented in practice and used as intended. However, our study reveals that there is still some work to do before PPC is understood and accepted by all those involved.

## Background

Children (0–18 years) living with life-threatening or life-limiting conditions is a growing patient group [1, 2] in need of complex health care services from a skilled PPC team [3]. In 2008, a group of paediatricians from Europe, Canada and the USA (International Meeting for Palliative Care in Children, Trento) published a consensus statement about comprehensive and integrative approaches recommended for these young patients, which should include active and comprehensive care for the child’s body, soul and mind and should involve the entire affected family [4]. This approach is based on the World Health Organization’s [5] definition of paediatric palliative care (PPC) and is recommended as the core standard of care for all children with LT/LL conditions in Europe [4].

The PPC population is characterized by a heterogeneous range of conditions with a higher proportion of non-cancer diagnoses compared to adult PC [2, 6, 7], and includes bereavement support and follow-up with the families after the death of the child [8]. The overall aim of PPC is to improve the quality of life (QoL) of the child and the child’s family by ensuring early and correct identification, assessment and treatment of pain and suffering, whether these are of a physical, psychosocial or spiritual nature [9]. In order to fulfil this holistic aim, interdisciplinary teamwork must be an integral part of PPC [10, 11].

Historically, the concept of PC developed from the field of adult oncology and has focused on alleviating suffering at the end of life [4, 10–12]. Due to demographic and technological changes, the concept of PC has evolved to address the needs of patient populations that may not be characterized as dying, but to whom relief of suffering and improvement of QoL are important [13, 14]. This is particularly relevant for children living with life-threatening or life-limiting conditions and their families. Across countries and due to different cultural background different terms are used to describe holistic, interdisciplinary, family centred care for children with serious- and/or life-threatening diseases [15, 16]. Differences in infrastructures- and delivery of health-and social care services, might as well explain differences in understanding of the concept of palliative care [13, 17]. Life-limiting and life-threatening conditions are terms often used to define the population of children who would benefit from follow up from paediatric palliative care services [2], and supportive care are used within oncology [18].

However, a new consensus-based definition of PC defines it as ‘the active holistic care of individuals across all ages with serious, health-related suffering […] which cannot be relieved without medical intervention, [and] which compromises physical, social, spiritual, and/or emotional functioning’ [13]. By presenting the process of achieving consensus, the authors [15] demonstrated the breadth of healthcare professionals’ (HCPs’) understanding of the term PC, which is often used interchangeably with hospice care, end-of-life care and terminal care [15].

In dictionaries and the literature, concept analysis has been applied to clarify the concept of PC [13, 18, 19], palliation [20], PC nursing [19] and PPC [21, 22]. Some sources define PC as care, while others understand it as counselling, treatment and/or accompaniment [19]. Meghani [14] identified four attributes of PC: total, active and individualized patient care; support for the family; interdisciplinary teamwork; and effective communication. Applequist and Daly [20] found that the concepts of palliation and PC have several similarities, but while PC involves provision of physical, emotional, psychological and social care, palliation is a patient-centred outcome, such as symptom relief by means of a noncurative intervention and human presence. One study found that palliation and alleviation are not synonymous concepts but that they are complementary [23]. HCPs describe the term PC as a blurred and confusing concept that is associated with end-of-life care [24]. The lack of clarity among HCPs about what constitutes PC causes patients to believe that death is imminent when PC is introduced [12]. The association with end-of-life care could create negative emotions among HCPs, relatives and patients [24]. A standard definition and a clear understanding of PC is important to clarify the concept and what it implies for HCPs and their patients. PPC is a relatively new discipline in Norway. In 2016, the Norwegian Directorate of Health published the first national guideline on palliative care for children and adolescents, providing guidance on education, organization, and practice of PPC in Norway [25]. This white paper is based on international and European standards for palliative care. The only postgraduate program (master’s degree level) in PPC in Scandinavia was established in 2017 at Oslo Metropolitan University, educating HCP to work in PPC-teams. Interdisciplinary PPC-teams have been established in children’s wards throughout the country providing care regardless of whether the child receive care at home, in institutions or in hospitals.

To our knowledge, no studies have investigated how multidisciplinary HCPs understand the concept of PPC. The aim of this study is to explore the concept of PPC from the view of HCPs in a paediatric setting. The research question is: How is PPC understood and used among HCPs involved in PPC?

## Methods

### Methods and data

The study employed an explorative and descriptive design with a qualitative method using focus group interviews. This design is suitable for exploring HCPs’ experiences. The group dynamics in focus groups may highlight different perspectives among the participants, enhance data quality and generate data that would not be found in individual interviews [26]. The reporting of the study was guided by the consolidated criteria for reporting qualitative research [27].

### Recruitment and sample

The participants were recruited from three paediatric units in two hospitals located in Eastern Norway. The units consisted of different departments with children from 0 to 18 years with different diagnoses. Contact persons at the units recruited the participants using purposeful sampling [26] according to the following criteria: health and social care professionals employed at a paediatric ward who worked with children in need of PPC, regardless of whether they were members of a multidisciplinary PPC team or not. Potential participants received oral and written information about the study. The final sample consisted of four focus groups with a total of 21 participants (see Table 1). Two participants were men, and 19 were women. Most of the participants had worked with children with life-threatening or life-limiting conditions for more than 10 years, and some for as long as 23 years. Four participants had less than 2 years of experience working with paediatric patients. Two participants worked as patient coordinators. At the time of the interviews only two participants had formal education in PPC. Social workers were included in the study as HCPs because they perform patient-oriented, clinical work. In one of the focus groups, four participants withdrew their consent prior to the interviews, which is the reason why this group had only two participants. No reason was given for the withdrawal.

**Table 1:** Participants

Interview Number	Number of Participants	Professions
Interview 1	4	Paediatric nurses (2) Nurses (2)
Interview 2	9	Clinical social worker Oncology nurse Paediatric nurses (2) Nurse Chief Physician Specialist in psychology Paediatrician Physiotherapist
Interview 3	2	Physiotherapist Paediatric nurse
Interview 4	6	Nurses (3) Clinical social worker Chief physician Medical doctor

### Data collection

The focus group interviews were conducted between November 2019 and February 2020 in a meeting room at the participants’ workplace. Each interview lasted from 45 to 90 min. The interviews were conducted by a moderator and an assistant moderator (AL, KR, LGK and EAF). All interviewers had an HCP background, and none had any personal or professional relationship with the participants. The interviewers informed the participants about the reasons for the study and the professional background of the research group. An interview guide was used to initiate dialogue and provide the focus for the discussion among the participants. The interview guide was developed based on findings from previous research. The interview guide was not piloted but was discussed within the research team and with a reference group, including user representatives, to ensure that the questions were relevant and clearly formulated. The guide contained open-ended questions related to the concepts of PC, PPC, alleviation and end-of-life care. In addition, the participants were encouraged to illustrate these concepts by sharing their experiences from working with these children and their parents. The interviews were audio recorded.

### Data analysis

The focus group interviews were transcribed verbatim by an external transcriber and were analysed using qualitative content analysis [28, 29]. The interviews were read several times to gain an understanding of the material as a whole. Thereafter, interviews were read line by line to identify meaning units. The meaning units were condensed and coded using descriptions based on the text. Guided by the research question, the codes were examined for similarities and differences across the interviews and then sorted and abstracted into categories in line with the manifest content. To develop themes, the categories were abstracted and interpreted, allowing disclosure of the latent content. An example of the analysis process is shown in Table 2.

**Table 2:** Example of the analysis process

Meaning unit	Condensed meaning units	Codes	Categories	Theme
‘Because this concept is very scary and it’s so [...] yes I think then [...] that I imagine maybe many parents think so too. Then you hear that, “ok, now we are in a palliative phase”, then [...] I think a lot of people can probably think, “ok then it’s death that’s the outcome, now we’ve got it”. [...] It cannot change then, it cannot be reversed. It’s a bit of a scary concept’.	The concept is scary. Many parents think so, too. Once hearing that the child is in a palliative phase, many likely think that death is the outcome. Once they have received that message, it cannot be changed.	Scary concept for both HCPs and parents, associated with death	A concept still associated with death and dying	**Frightening concept that evokes negative emotions**
‘But for many, the fact that we mention the word palliation will still be completely dramatic, that we could risk them disappearing out the door’.	Mentioning the word ‘palliation’ can be dramatic, and there is a risk of losing parents.	Dramatic word, risk that parents are lost	HCPs’ responsibility for introducing and using the concept and obtaining a common meaning	
‘But that term palliation [...] I do not think we mentioned it today. And I do not feel like it either. Because I think it’s a concept that [...] I envision a cancer ward with completely terminally ill cancer patients. In my head, it’s like that, sorry’.	The concept of PC is associated with cancer wards and terminally ill cancer patients.	PC associated with terminal illness	A concept still associated with death and dying	

Researcher triangulation was used to facilitate credibility [30]. Each step of the analysis was conducted independently by AW and SAS, who then discussed each step and reached an agreement by consensus. Furthermore, the second author asked critical questions to enhance alternative interpretations and movement beyond the preconceptions. The categories and themes were nuanced and corroborated through discussions among the research group, which consisted of eight women and one man.

### Ethical considerations

The Norwegian Centre for Research Data (reference number 935944) and the local data protection officers/ethical board at the two hospitals approved the study (reference number 19/21909 and 21/10389). Both NSD and the local protection officers has the authority to assess whether ethical principles have been safeguarded in projects that do not fall under the Health Research Act. Since study participants are healthcare professionals, and the project does not collect data about health and illness the project did not require permission from a regional committee for research ethics. The project meets all necessary ethical requirements and guidelines. The participants were informed that their participation was voluntary, that anonymity and confidentiality would be safeguarded and that they could withdraw at any time without giving any reason. Written informed consent was obtained from the participants prior to the focus group interviews.

## Results

Two themes emerged from the data analysis: *a frightening concept that evokes negative emotions* and *a broad and complementary concept* (Table 3).

**Table 3:** Overview of themes and categories

Themes	Frightening concept that evokes negative emotions	Broad and complementary concept
**Categories**	An unfamiliar and unmeaningful concept	Total care for the child and the family
	A concept still associated with death and dying	Making room for life and death at the same time
	HCPs’ responsibility for introducing and using the concept and obtaining a common meaning	The meaning of alleviation and PC

### Frightening concept that evokes negative emotions

#### An unfamiliar and unmeaningful concept

Some participants indicated that they were unfamiliar with the concept of PPC. Several of them said that the concept was not widely used within paediatric oncology, neurology or towards children with progressive conditions: ‘I simply agree with what has been said already, that palliative care is supposed to be the concept […] I don’t think I’ve ever used that concept when talking with parents’ (Interview 2, Participant 7). Participants in all the focus groups used cancer as an example of the kind of illness in which children would need PPC.

Participants working at Departments of Paediatric Neurology explained that even though the concept of PC included the services they actually provided to children and families, they did not use the term PPC in communication with families. While some participants described having conversations about QoL and treatment limitations with families, other participants working at Department of Paediatric Neurology felt that PPC was not a meaningful concept to them. These HCPs who were caring for children with neurodegenerative diseases and neurological conditions, also stated that they would use the concept of PPC if there was a common understanding among HCPs about the meaning of this concept, including pain relief and total care of the child and family.

#### A concept still associated with death and dying

Participants associated PPC with death and dying, diagnoses with shortened life expectancy, terminal care and premature death. Across the focus groups, participants described PPC as a frightening concept due to these associations, and they assumed that it would frighten parents as well. When describing the concept of PPC, the participants used words and phrases such as ‘scary’, ‘burdensome’, ‘death’, ‘dramatic’, ‘limiting’ and ‘absence of active treatment’. However, one participant said that PC had positive connotations, referring to the potential understanding of the concept as ‘to be cared for’.

Several of the participants stated that PPC is ‘not active treatment’ and that PC begins when medical treatment ceases. Others expressed that ‘it’s all about care’, associating PC with more than death. One participant recalled having learned in nursing school that PC was equal to care of the dying. While elaborating on this, the participant and the other group members realized that the present concept of PPC is no longer limited to death and dying. The participants also discussed challenges that could arise if this concept is used from the time of diagnosis and throughout the illness trajectory, even during periods when the child is not suffering: ‘It’s challenging when the meaning of a concept changes, from being related to a specific phase of illness, to encompassing an entire childhood […] even from the time when there were no problems, really’ (Interview 3, Participant 1).

The participants found it challenging how different conditions and illness trajectories of children in need of PPC could last from 1 to 10 years or even be lifelong, and they indicated that children waiting for surgical treatment or recovering after a surgical procedure may no longer be considered to be in need of PPC. These discussions raised other associations than those of death and dying, such as when the term PPC should be used and provided.

#### HCPs’ responsibility for introducing and using the concept and obtaining a common meaning

The unpredictable illness trajectories of some children made it difficult for the participants to foresee when a child was going to die or if a child would have a long life. This uncertainty made it challenging for participants to use the concept of PPC and to provide sufficient information to the families. Participants stated that not all parents were willing to talk about difficult matters regarding their child’s illness, and they discussed the importance of HCPs being open and ready for such talks with the child and/or the parents when they seemed interested in or receptive to those conversations. However, one participant also said that it seemed more difficult to talk about the death of a child than an adult, which affected the timing for introducing PPC: ‘I believe we have a larger barrier to start talking about death and dying in children than in adults […] I think we are waiting much longer’ (Interview 2, Participant 6).

The participants perceived the passing of time to be essential for parents to accept their child’s condition. They suggested that the concept of PPC should not be introduced before the parents had started accepting their child’s life-threatening or life-limiting condition. The participants discussed the uncertainty about whether parents are familiar with this concept and whether PPC is regarded as an option for their child. The participants feared that if they used the term PPC in their communication with families, it could evoke strong negative emotions and thus threaten the trusting relationship between the care team and the parents. As a result, participants said that PPC was seldom introduced early in the illness trajectory. In contrast, other participants underlined that introducing PPC at an early stage could be important to broaden parents’ understanding of this concept and what it could mean to them:

I think it is important that we, the HCP, use the concept [of PPC] and thereby make it less frightening. If we are unable to use the word ‘palliative’, it will be even harder for the parents to come to terms with it. (Interview 1, Participant 4)

Likewise, it may take courage to talk about death and dying, and the participants underscored that a mutual understanding and agreement about the concept could make the care team more at ease with the concept: ‘Talking about it with each other [HCPs] will perhaps make it easier for us as well, and we may feel more confident in such a situation’ (Interview 2, Participant 8).

Similarly, the participants’ need for a common understanding of PPC was regarded as important in communication not only within the care team. Some felt that it could be easier to use this concept if it was better known by the general public, as described by one participant:

I find it difficult that this concept is meant to be so all inclusive when the general public has not yet accepted it this way. That’s why we always must consider: can I use this word now or not? The worst possible outcome would be [...] if the dialogue with them [the parents] is lost. We would never take such a risk. (Interview 3, Participant 1)

A common understanding of the concept of PPC between HCPs and parents could enhance HCPs’ ability to provide flexible care for the child and family. The participants underlined that it is the HCPs’ responsibility to explain to the parents what the concept of PPC means. For that to happen, HCPs must take ownership and use the term themselves. Further, the participants across the focus groups agreed that since PPC has negative connotations, HCPs might need to be encouraged to use the concept in communication with the children and the parents and should collaborate with other HCPs to promote a common understanding of the concept.

### Broad and complementary concept

#### Total care for the child and the family

The participants stated that PPC is about total care that uses an interdisciplinary approach and includes the whole family, emphasizing child participation and taking care of each family member’s individual needs. Interdisciplinary collaboration was perceived as collaboration between the family and HCP as well as with other professionals like hospital clowns, priests and music therapists and with institutions such as schools, kindergartens and local community resources. In this collaboration, the participants highlighted how much they valued the support from specialized PPC teams.

The participants perceived that every stage of a child’s PC trajectory has the same goals: optimized treatment and QoL. However, the achievement of these goals depends on the child’s condition and individual needs. The participants described how supporting the needs of a child could mean helping the parents manage their child’s pain and discomfort, helping children and families verbalize their experiences or to describe the physical changes that the child is undergoing. Participants expressed that it was crucial to help parents to see beyond the illness, to provide hope and to focus on the healthy aspects of the child’s development and skills (and not their lack of skills). The participants stated that they strove to do their very best to provide the child with the best possible support, for instance, by talking about other things that were important to the child rather than focusing on their disease.

#### Making room for life and death at the same time

Reflecting on their understanding of the concept of PPC, participants spoke about the demanding situation for parents not knowing when their child might die and the issues they have to deal with: ‘They have to make room for several things at the same time, making room for life and death at the same time and make room for joy and grief at the same time’ (Interview 2, Participant 7).

The uncertainty among parents regarding when their child might die was perceived by the participants as demanding, and HCPs indicated that those parents expressed feelings like sorrow, fear and guilt. One participant illustrated this:

A mother who worried that her child would suddenly die of an infection, she always kept some of the child’s dirty laundry, so she could keep the smell of her child in case something suddenly happened during the night. She does this, while at the same time, the child is going to school, and the child’s life is filled with joy. [...] If I fulfil the child’s wish and we go swimming, what if the child dies, will it be my fault, as a parent? Will I be responsible for the death of my child? Living with this risk, living with these questions about what the child can handle, and what the child needs to live a meaningful life – I think this is something that should be addressed early in the course of a life-threatening and unstable condition, even if the child may live for years. Something could suddenly happen. (Interview 2, Participant 7)

Participants highlighted that PPC was about preparing parents for the challenges to come and that information and conversations between HCPs and families about how to facilitate the best possible QoL for the child must start early in the PC trajectory.

#### The meaning of alleviation and PC

Participants preferred the concept of alleviation rather than PC, especially in their communication with children. This preference was based on the assumption that children understand the word ‘alleviation’ because it is something they experienced with their own body.

Some participants associated the word alleviation with end-of-life care, when pain and symptom management is of paramount importance, while others thought that alleviation was about promoting QoL. There was some disagreement about the timing of alleviation in an illness trajectory. Some thought that the palliative phase lasts for a long period, and alleviation begins when the child is dying, while others perceived alleviation in a broader sense that is not restricted to end-of-life care. While reflecting on timing, one participant said that alleviation is what takes place during wound care or dressing change, or when the family needs support. The concept of alleviation was discussed as not only pertaining to physical needs, as it can also imply reconciliation with pain or sorrow that cannot be erased. Even playing can be alleviating and a form of relief. According to the participants, alleviation is also about the total care of the child and the family as described in the previous category. Moreover, the participants underpinned that the goal of PPC is alleviation regardless of timing since PC may last for a long time or, for some children, their entire lives. Different phases throughout illness trajectories were described to have different attributes, for example, some participants expressed that there is still hope while the child receives alleviation, while there is ‘no longer any hope in the terminal phase’ (Interview 1, Participant 3). For many participants, providing hope was viewed as part of PC and the alleviation of suffering.

## Discussion

The aim of this study was to explore the concept of PPC from the view of HCPs in a paediatric setting. Our analysis identified two themes regarding PPC: that it is a frightening concept that evokes negative emotions and a broad and complementary concept.

Our participants viewed PPC as an unfamiliar and unmeaningful concept and referred to associations with death and dying, using descriptions such as scary, soaring, burdensome, dramatic and lack of active treatment. Despite being a misconception [9], such associations are in line with recent research findings showing that PC is still associated with cancer and the final weeks of life [13, 24]. These associations regarding PPC, often accompanied by negative emotions, might be rooted in earlier professional career experiences but could also be culturally conditioned. HCPs’ memories of patients’ deaths that occurred during training or their early careers could cause feelings of helplessness, guilt or ongoing stress, which may have a lasting impact on their professional and personal lives [31]. However, HCPs’ ability to cope with stressful events is individual and affected by their personality [32]. Education programmes have been found to reduce the cultural taboo for HCPs surrounding the topic [33], and HCPs receiving formal grief and bereavement training are more comfortable discussing death with families [34]. Awareness of these issues together with supportive structures might strengthen HCPs’ ability to handle difficult situations in PC.

One study found that HCPs who discussed children’s end-of-life care with colleagues tended to feel more comfortable interacting with and initiating discussions with families about a child’s death [34]. Similarly, we witnessed a development in the views on PPC during our focus group interviews. In one of the focus groups especially, the participants asked each other questions and reflected together on the meaning of PPC. This suggests that HCPs’ understanding of the concept of PPC is a dynamic process and that increased awareness contributes to the development of the concept and its content. Learning in groups might lead to a collective understanding or a consensus about the concept and development of PPC. This could prove particularly important, as a lack of mutual understanding has been found to negatively affect communication about poor prognoses in childhood cancer [35].

It is well-known that HCPs use words like ‘hospice care’, ‘end-of-life care’ and ‘terminal care’ interchangeably with PC [15]. Wallerstedt et al. [24] found that HCPs perceived PC as a blurred and confusing concept and that they preferred using words like ‘terminal care’ and ‘last days life’ rather than PC. Further, previous studies have suggested that there is strong stigma attached to the term ‘palliative’ [36, 37]. Using other words, such as ‘kindness and love’, ‘peace’, ‘religion’ and ‘supportive care’ have been proposed as a strategy to reduce the negative perceptions associated with PC [36, 37].

Lack of knowledge and negative connotations can hinder integration of PPC [24, 38], and both education and clinical experience are vital for increasing the attention given to, competence with and integration of PPC [39–41]. Most of our participants preferred using the concept of alleviation to PPC because they found alleviation to be a broader concept that carried fewer negative connotations. Thus, using the word ‘alleviation’ might be more acceptable or comfortable for both parents and HCPs.

Some of our participants discussed their experience that parents instantly associated PPC with death and thought their child would die when this concept was used. Consequently, these participants avoided using PPC because they were afraid of losing a good relationship with and trust from the parents. Previous research has shown that HCPs working in the field of oncology find it important to have regular conversations about treatment goals from an early stage and to be open and honest when communicating about the end of life [42]. The use of a screening instrument might improve the timely identification of children that could benefit from PPC and consequently introduce PPC in an early stage [43]. Even though guidelines for facilitating communication regarding PPC exist, HCPs find it difficult to discuss matters related to death dying with colleagues [44]. Parents, on the other hand, have reported insufficient or poor information related to issues about their ill child, and some do not dare to ask questions to clarify these issues [45]. This may be due to HCPs’ reluctance to discuss challenging issues with the families or HCPs’ fear that this kind of information might take away hope. HCPs may believe that the families are not ready to receive such information and claim that difficult conversations deserve and demand uninterrupted time, which they lack [45]. Lövgren et al.’s (2021) findings aligned with our own, suggesting that a mutual relationship based on trust is crucial and that the perception of PPC as an unfamiliar concept might threaten this trust. Open and honest communication is a prerequisite for trust [35, 46]; however, communication challenges, emotional and mental drain, lack of mutual understanding and insecurity regarding communication skills are challenges experienced by HCPs in their communication with families of seriously ill children [35]. Training in communication and receiving communication support from colleagues [35] could substantiate trust, which is important as trust is a core element for parents of seriously ill children and highly valued in their communication with HCPs [47].

Even though participants across disciplines in our study did not use the concept of PPC very often, they highlighted that they considered themselves responsible for the content and meaning of PPC. Further, they described the core elements of PPC in compliance with previous research, namely as the total care of the child and the child’s family using an interdisciplinary approach that is aimed at improving QoL [48].

Our participants underpinned that PPC is about focusing on whatever gives joy and the best possible QoL for the child and the child’s family, which is in line with previous descriptions of PPC [8, 11]. Further, our findings suggest that the participants considered it crucial to make room for both life and death at the same time. The story told by one of the participants about the mother who worried that her child would suddenly die while at the same time, the child’s life was filled with joy. The story indicates that HCPs can understand the very demanding dilemmas faced by parents, who must balance a fear of their child’s death with their wishes for their child to live as normal of a life as possible. When a child becomes more vulnerable through illness or disability, the role and value of play increases. Therefore, it is important to support play and everyday life as long as possible through facilitation and pain relief [49]. Interaction with peers is important for all children [50] and especially for the QoL of children in PC [10]. Even children living on mechanical ventilation can interact with peers through social media or home visits from peers [50, 51].

Our participants highlighted hope as important throughout the illness trajectory; nevertheless, some believed that there was no hope when the child reached the terminal phase. When associating death with the concept of PC, the participants found this to be in conflict with the need to keep up hope. To parents of seriously ill children, keeping up hope is important for their coping with the situation. Yet, the meaning of hope is not a static state but something that may change throughout the illness trajectory [52]. For parents, hope might not be limited to hope for a cure or treatment response [53] but might also include hope for a good QoL, spiritual and physical wellbeing, a peaceful death without pain and meaningful relationships [52–54]. HCPs can support parents by understanding their needs [35], but to do so, and because the meaning of hope changes over time, they should regularly talk with parents about their current hopes [52]. Thus, hope has its natural place in PPC, as it might provide support and guidance for both parents and HCPs, ensuring that all strive to secure the child’s QoL. Nevertheless, communicating hope while at the same time making parents aware of the reality of a poor prognosis is a challenging task [42]. Our study revealed that the term palliative care is complicated and that there is a need for more information and discussions about what PPC really means. Collaboration between patients, relatives, patient organization, HCP and researchers could be important to facilitate discussions about PPC earlier in the illness trajectory and to enhance HCPs ability to address issues that are important for children and families. PPC teams can play a key role in making PPC better understood by teaching and supervising families and other HCPs about PPC. In Norway PPC-teams are established, but the teams are still lacking sufficient recourses to employ HCP from different disciplines in full time positions to established robust teams who can support both children, families, and other HCPs. Better legal and financial structures would improve both quality of care and access to care [10].

### Strengths and limitations

The authors have varied clinical and research expertise in PPC and adult PC, and several have expertise in qualitative research. The composition of the researchers who both conducted the interviews and who performed the analyses was an important strength and enriched the nuance in the analyses.

Participants shared sensitive and positive and negative experienced regarding PPC. They felt safe, which likely contributed to their stories and discussions. A limitation may be that the participants were from only two different institutions in the same geographically region and only from a hospital setting. Focus groups from hospitals in other parts of the country or HCPs from municipal PPC might have other or different experiences and could possibly enrich the material. Another limitation could be that one of the focus groups included only two participants and could have limited the experiences with PPC share in this interview. However, in a smaller group each participant can have more time to talk about their experiences.

## Conclusions

The HCPs in this study understood PPC differently, but most of them highlighted QoL, total care for the child and the child’s family and interdisciplinary collaboration as core elements. Several still associated PPC with children with cancer, even though they were familiar with widely used definitions and even though they worked with children with other diseases. Even so, they included children with other diseases in their discussions as well.

Some respondents associated PPC with death and dying, and several were unfamiliar with the term PPC. They were afraid of losing trust with parents by introducing the term too early in an illness trajectory. In contrast, participants emphasized the importance of focusing on life, facilitating play and emphasizing on the healthy aspects of the child’s life. The results gave the impression that most participants preferred the term ‘alleviation’ to ‘PPC’.

PPC is mostly used when medical treatment ends, and the term is often used synonymously with end-of-life care and terminal care. Participants highlighted that it is the responsibility of HCPs to understand the concept and to introduce it to families in a way that the families can comprehend.

Attention to and knowledge among HCPs might change the perception about PPC from a frightening concept to one that is accepted by all parties, implemented in practice and used as intended. However, our study revealed that there is still some work to be done before PPC is understood and accepted by all those involved.

## Data Availability

The data transcripts and analysis are not publicly available due to privacy and ethical concerns. Data is collected for this specific research project, and according to Norwegian law, and in accordance with Regulation (EU) No. 2016/679 (General Data Protection Regulation), data can only be processed within the purpose stated to the Data Protection Officers, and as stated in the informed consent. The informed consents are given to the data Controllers at the hospitals where data were collected. Data can only be used in accordance with the stated purpose and handing over data to journals in order to submit a publication would be contrary to Norwegian law.

## Abbreviations

PC: Palliative care
PPC: Paediatric palliative care
HCP: Healthcare professional
LT/LL: Life-threatening/life-limiting
QoL: Quality of life
